# Intercellular transfer of P-glycoprotein mediates the formation of stable multi-drug resistance in human bladder cancer BIU-87 cells

**DOI:** 10.1242/bio.041889

**Published:** 2019-04-09

**Authors:** Xiao-Zhi Cheng, Hui-Liang Zhou, Song-Xi Tang, Tao Jiang, Qin Chen, Rui Gao, Yi-Lang Ding

**Affiliations:** 1 Department of Urology, First Affiliated Hospital, Fujian Medical University, Fuzhou 350005, People's Republic of China; 2 Department of Urology, Huanggang Central Hospital, Huanggang 438000, People's Republic of China

**Keywords:** Bladder cancer, Multidrug resistance, P-glycoprotein, Intercellular transfer

## Abstract

We investigated the biological characteristics of acquired drug-resistant cells (AqMDRs) formed by intercellular P-glycoprotein (P-gp) transfer and whether AqMDRs can form stable drug-resistant strains. Drug-sensitive BIU-87 cells were co-cultured with doxorubicin (DOX)-resistant derivative BIU-87/DOX cells in transwell chambers for up to 96 h. The presence of P-gp in recipient cell membranes (AqMDRs) was detected by confocal microscopy, CCK-8, western blot, and RT-PCR were used to detect resistance index (RI), P-gp expression and *MDR1* mRNA expression in AqMDRs after 0, 4, 8, 16, and 20 passages and frozen/resuscitated twentieth generation AqMDRs. There was an increase in P-gp transfer with longer co-culture times of drug-resistant and sensitive strains. Without DOX, although the AqMDR numbers increased with each passage, the RI and P-gp expression decreased gradually, and the expression level of MDR1 mRNA did not change significantly. With DOX, the RI and P-gp expression increased slightly, and the MDR1 mRNA expression level gradually increased to the BIU-87/DOX level. AqMDRs can grow stably at drug concentrations slightly higher than the IC50 of sensitive strains, which sensitive strains cannot survive. P-gp transfer between cells gradually increases with longer co-culturing of drug-resistant and sensitive strains. The drug resistance of AqMDRs decreases without drug intervention, but with drug intervention, cells can maintain resistance and gradually develop into stable drug-resistant cells.

This article has an associated First Person interview with the first author of the paper.

## Abstract (precis)

**Summary:** Intercellular transfer of P-glycoprotein forms stable multi-drug resistance, which complements the formation mechanism of MDR and indicates that new treatment strategies need to be designed to overcome MDR.

## INTRODUCTION

P-glycoprotein (P-gp) encoded by the human *MDR1/ABCB1* gene, is a cell membrane glycoprotein with a molecular weight of 170 kDa, first discovered in drug-resistant Chinese hamster ovary cells in 1976 by Juliano and Ling (1976). As an energy-dependent drug pump, P-gp is powered by ATP, pumps intracellular drugs out of the cell, thereby reducing the intracellular drug concentration, which is not only a self-defence protection mechanism under physiological conditions in the body but also one of the main causes of tumour multidrug resistance (MDR).

MDR is also common in bladder tumours, and expression of the MDR1 gene can be detected in bladder cancer tissues from more than 75% of patients (Featherstone et al., 2005). The effective rate of simple chemotherapy for bladder cancer is only 51.5% (Sylvester et al., 2005). Moreover, the expression of P-gp was negatively correlated with the prognosis of bladder cancer (Hoffmann et al., 2010). Our previous studies (Zhou et al., 2013) have shown that P-gp can also be transferred from the resistant bladder cancer cell line BIU-87/DOX to the sensitive strain BIU-87, which is not resistant to doxorubicin (DOX), providing the first evidence that this ‘non-genetic’ acquired MDR mechanism also exists among bladder cancer cells.

Pasquier et al. (2012) found that the degree of P-gp transfer between cells was positively correlated with the time of co-culture. Importantly, as the culture time is prolonged, the activity of P-gp also gradually increases. Our previous study (Zhou et al., 2013) used a rhodamine 123 efflux test to initially confirm that bladder cancer acquired drug-resistant cells (AqMDRs) functions via drug efflux. However, the development and outcome of AqMDRs have not been further studied. In the present study, we investigate the biological characteristics of AqMDRs formed by intercellular P-gp transfer and whether AqMDRs can form stable drug-resistant strains.

## RESULTS

### Observation of AqMDR cells by laser confocal microscopy

**Fig. 1.**
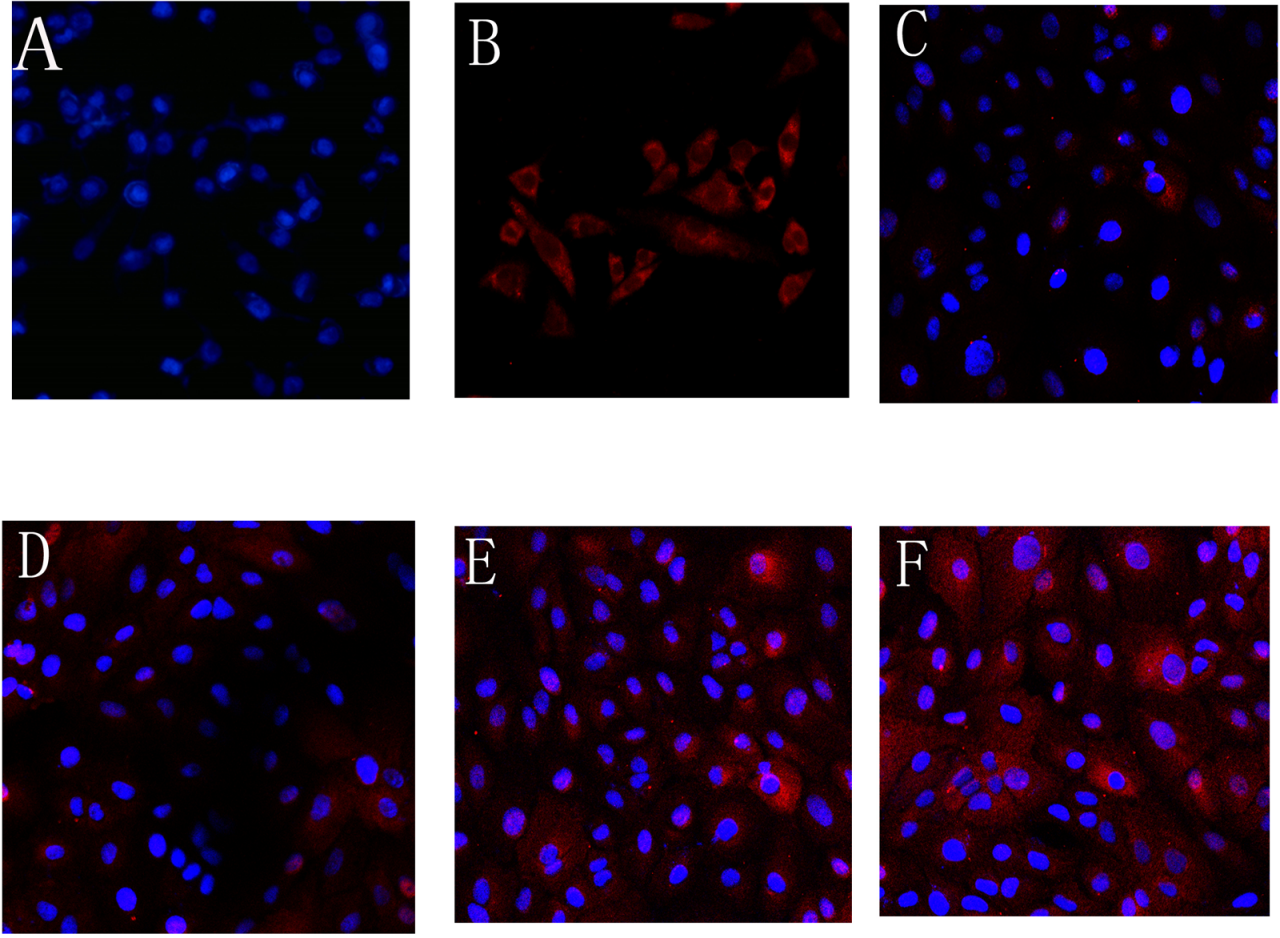
**P-gp content of AqMDR cells observed by laser confocal microscopy (×200).** BIU-87 (A) and BIU-87/DOX (B) cells were co-cultured in transwell chambers for 24 h (C), 48 h (D), 72 h (E), 96 h (F), TR-labelled goat anti-mouse IgG indirectly labelled P-gp (red fluorescence), Hoechst 33342 labeled AqMDR nuclei (blue fluorescence).

BIU-87 and BIU-87/DOX cells were co-cultured in a transwell system for 24 h, 48 h, 72 h, or 96 h. The cells (AqMDR), whose nuclei were stained with bright blue fluorescence by Hoechst 33342, displayed red immunofluorescence staining of P-gp, mainly in the cell membrane and cytoplasm (see Fig. 1). Fig. 1 shows that P-gp is uniformly distributed from the initial few cells to 96 h, indicating that the P-gp transfer increases with the increase in co-culture time.

### Cell growth curve

**Fig. 2.**
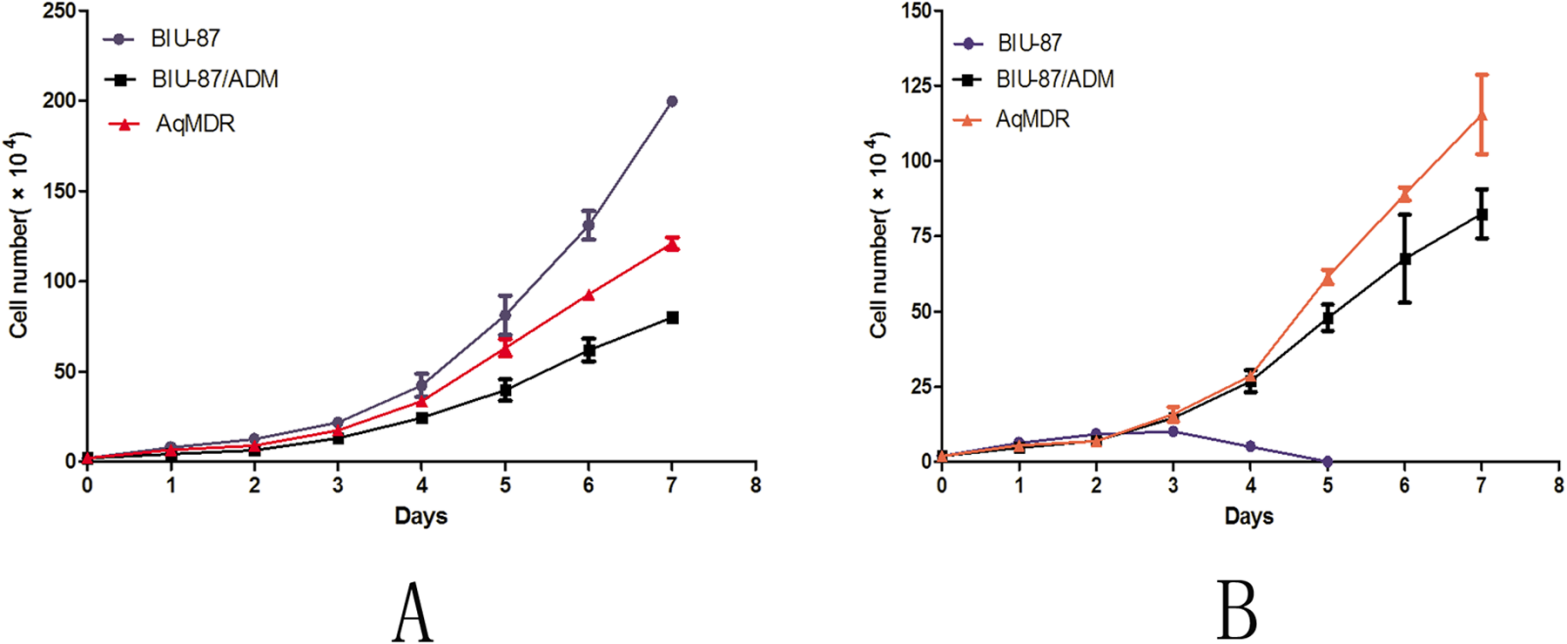
**Cell counting assay for cell growth curves.** Without DOX (A) and with 1 μg ml^−1^ DOX (B).

BIU-87 doubling time (25.30±0.04 h) was shorter than that of BIU-87/DOX (31.58±0.37 h) (*P*<0.001), and that of AqMDR was between the two (28.39±0.33 h, *P*<0.001), as shown in Fig. 2A. In the 1 μg ml^−1^ DOX group, growth inhibition was observed after 3 days of BIU-87 culture. Large areas of adherent cells detached, and all died after 5 days, while AqMDR cells were not restricted in growth, and their doubling time was shorter than that of BIU-87/DOX. (28.76±0.78 versus 31.35±0.81, *P*<0.05), as shown in Fig. 2B.

### RI of AqMDR cells in different treatment groups

Without DOX group, the number of AqMDR cells decreased with each passage, and the RI gradually decreased (r=−0.988, *P*<0.001). After 1 month of cryopreservation, there was no significant difference in the IC50 between the resuscitated AqMDR cells and the BIU-87 sensitive strain cells (0.779±0.007 versus 0.859±0.015, *P*>0.05). In contrast, with DOX group, the AqMDR cell number increased with each passage. The RI increased gradually (r=0.954, *P*<0.001), and the differences between the groups were statistically significant (*P*<0.05) (Table 1).

**Table 1.**
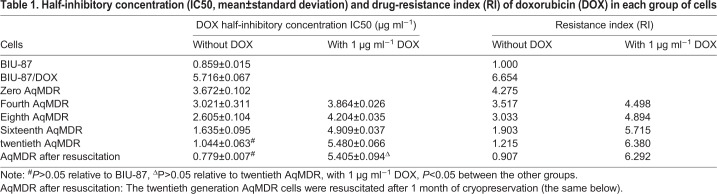
**Half-inhibitory concentration (IC50, mean±standard deviation) and drug-resistance index (RI) of doxorubicin (DOX) in each group of cells**

### Western blot analysis of P-gp expression in each group

**Fig. 3.**
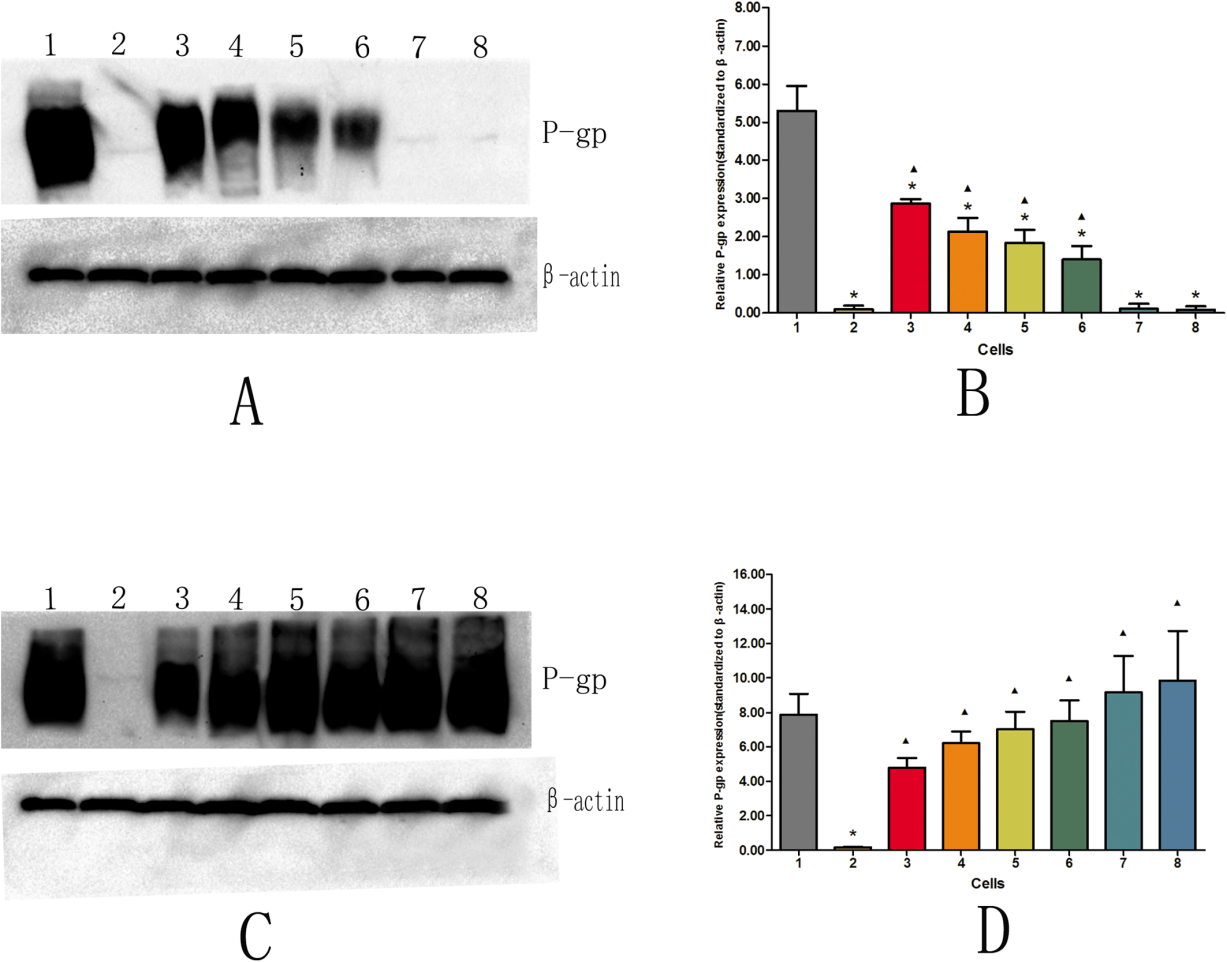
**Western blot analysis of P-gp expression levels in cells without and with DOX culture.** 1, 2, 3, 4, 5, 6, 7, and 8 represent cells BIU-87/DOX, BIU-87, zero generation AqMDR, fourth generation AqMDR, eighth generation AqMDR, sixteenth generation AqMDR, twentieth generation AqMDR, and AqMDR after resuscitation, respectively. (A,C) P-gp was detected by western blot, β-actin was used as an internal reference. (B,D) P-gp relative expression (P-gp gray value/β-actin gray value, *x̄* ± S). *P<0.05 with respect to BIU-87/DOX, ^▴^P<0.05 with respect to BIU-87.

Without DOX group, the P-gp expression level in AqMDR cells decreased gradually with increasing passages (r=−0.937, *P*<0.001). The difference was statistically significant (*P*<0.05) compared with P-gp expression in the resistant strain BIU-87/DOX (5.300±0.647). However, compared with the sensitive strain BIU-87 (0.089±0.094), AqMDR cells were passed to 20 generations (0.109±0.126), and the expression level of P-gp was not significantly different (*P*>0.05), as shown in Fig. 3A,B. With DOX group, the P-gp expression level of AqMDR cells did not decrease with increasing passage, although it increased slightly (r=0.818, *P*<0.001), and was not significantly different from the drug-resistant cell line BIU-87/DOX (7.880+1.198) (*P*>0.05). There were significant differences between the AqMDR cells and the susceptible BIU-87 cells (0.177±0.034) (*P*<0.05) (Fig. 3C,D).

### The mRNA expression of *MDR1* in each group was detected by RT-PCR

**Fig. 4.**
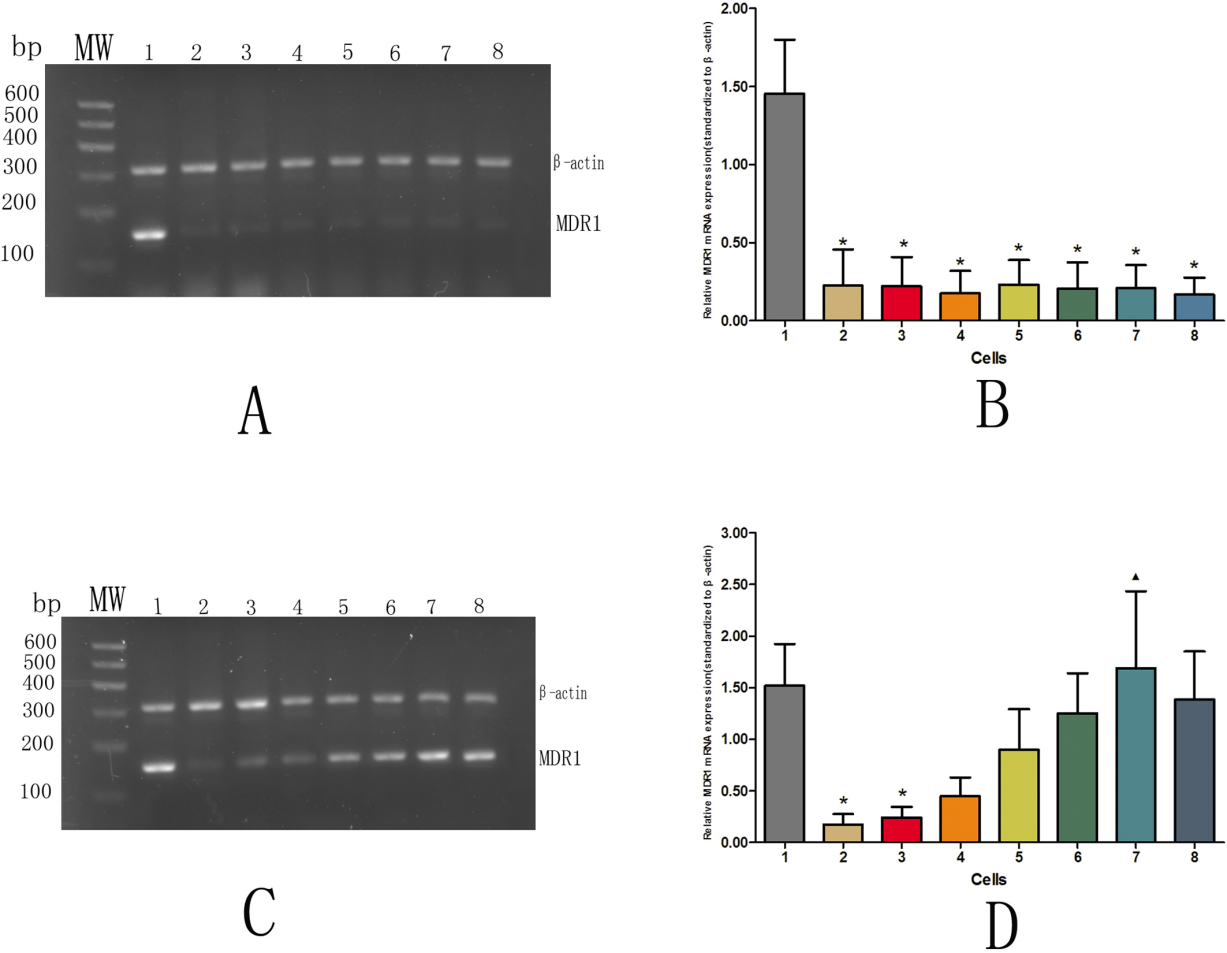
**mRNA expression levels of *MDR1* in cells cultured without and with DOX by RT-PCR.** 1, 2, 3, 4, 5, 6, 7, and 8 represent cells BIU-87/DOX, BIU-87, zero generation AqMDR, fourth generation AqMDR, eighth generation AqMDR, sixteenth generation AqMDR, twentieth generation AqMDR, and AqMDR after resuscitation, respectively. (A,C) Agarose gel results of PCR products, MW is DNA marker, and β-actin was used as an internal reference. (B,D) *MDR1* mRNA relative expression (MDR1 gray value/β-actin gray value, *x̄* ± S). * *P*<0.05 relative to BIU-87/DOX, ^▴^*P*<0.05 relative to BIU-87.

Without DOX group, the expression level of *MDR1* in AqMDR cells did not change significantly with increasing passages (r=−0.09, *P*>0.05). There was no significant difference in *MDR1* expression in BIU-87 cells (0.223±0.234) between different generations of cells (*P*>0.05). Compared with the drug-resistant cell BIU-87/DOX (1.452±0.346), the difference was statistically significant (*P*<0.05) as shown in Fig. 4A,B. With DOX group, *MDR1* expression in AqMDR cells increased gradually with increasing passages (r=0.825, *P*<0.001), and *MDR1* expression in the twentieth generation cells (1.688±0.745) was correlated with that in the sensitive strain cells BIU-87 (0.173±0.103). Furthermore, the difference was statistically significant (*P*<0.05). There was no significant difference between AqMDR cells and drug-resistant BIU-87/DOX cells (1.518±0.405) after the fourth generation (0.447±0.184) (*P*>0.05) (Fig. 4C,D).

## DISCUSSION

In this study, prolongation of the co-culture period increased the P-gp content on the surface of the AqMDR cells, indicating that the amount of P-gp transfer between cells was positively correlated with the time of co-culture. Therefore, we selected AqMDR cells co-cultured to 96 h to study their biological characteristics. The cell growth curve suggested that AqMDR cells could stably grow in culture medium containing 1 μg ml^−1^ DOX, a concentration that caused the gradual death of the sensitive strain BIU-87, thus indicating that the functional P-gp obtained by AqMDR cells protected AqMDR cells so that they were not killed. However, this protection is not permanent. In a drug-free medium, the P-gp content of AqMDR cells gradually decreased, and the MDR characteristics were gradually lost, indicating that the transfer of protein levels alone did not enable cells to maintain permanent MDR characteristics. It was thus concluded that AqMDR cells gradually died in culture medium containing 1 μg ml^−1^ of DOX. However, this is not the case. The P-gp content of AqMDR cells in DOX-containing culture medium did not decrease significantly. Further research on *MDR1* mRNA revealed that its expression level gradually increased. The cells upregulate *MDR1* mRNA expression by themselves, maintain MDR characteristics and survive. We believe that the upregulation of *MDR1* mRNA may be related to the role of DOX. In the process of doubling AqMDR cells in 1 μg ml^−1^ DOX medium, the decrease in P-gp content and the accumulation of intracellular DOX reached a dynamic balance, which promoted the upregulation of *MDR1* mRNA expression by DOX. This mechanism may be related to P53, *ras* gene mutation (Chin et al., 1992) and hypomethylation of the *MDR1* promoter region (Tada et al., 2000).

Bladder tumour tissue itself can inherit P-gp from its normal source tissue to form primary resistance (Tada et al., 2000; Diestra et al., 2003). Our results from western blotting and RT-PCR showed that the weak band of BIU-87 cells also confirmed this. This finding indicates that the expression of the *MDR1* gene does not need to be synthesized *de novo* under the action of chemotherapeutic drugs, which is more conducive to the formation of secondary drug resistance. We successfully induced the human bladder cancer-resistant DOX cell line BIU-87/DOX by using a drug concentration-increasing method for 8 months with reference to Guo (Guo et al., 1997). In this study, AqMDR cells gradually formed stable BIU-87/DOX cells in culture medium containing 1 μg ml^−1^ DOX but took only 4 months. The mechanism is consistent with the drug concentration-increasing method: during the doubling of AqMDR cells, the P-gp content is gradually reduced, the drug efflux function is weakened, and the intracellular DOX concentration is gradually increased. The process of increasing the concentration of the drug is well simulated because the stimulation is started at a higher concentration in order to achieve stable drug resistance in a shorter time.

This method also provides us with new ideas for establishing MDR cell lines, although with the development of biotechnology, the use of *MDR1* gene eukaryotic transfection technology (Aksentijevich et al., 1996) has not required such a long time to establish MDR cell lines. However, the traditional chemotherapeutic drug concentration gradient increase (Guo et al., 1997) and high-dose drug shock methods (McGovern et al., 1988) can better simulate clinical chemotherapy, and the biological characteristics of MDR cell lines will more closely represent clinical conditions.

In the process of tumour chemotherapy, a variety of MDR formation mechanisms coexist such that drug-resistant cells, sensitive cells and acquired drug-resistant cells coexist with different MDR expression levels (Knaust et al., 2000). Under the action of antitumor drugs, the drug-resistant cells transfer P-gp to sensitive cells so that sensitive cells are not easily killed by drugs, and there is enough time to upregulate the expression of *MDR1*, eventually forming resistance and thereby enhancing the overall resistance of tumour levels. Therefore, blocking the transfer of P-gp between cells will increase the sensitivity of chemotherapy. Previous studies (Pasquier et al., 2012; Bebawy et al., 2009) have found that intercellular P-gp transfer is mediated by microparticles (MPs) and tunnelling nanotubes (TNTs). Our previous studies (Zhou et al., 2013) have also indirectly demonstrated that MPs are involved in intercellular P-gp transfer. Although there is no literature on blocking intercellular P-gp transfer, cytochalasin B has been found to be able to block the formation of TNTs and block the transfer of substances between cells (Bukoreshtliev et al., 2009), LMP-420 and thiol panthioethylamine inhibit MPs release (Penet et al., 2008; Wassmer et al., 2005), and amiloride, cytochalasin D and similar compounds can inhibit the uptake of MPs (Escrevente et al., 2011), all of which provide ideas for blocking the intercellular transfer of P-gp.

There are still some shortcomings in this experiment: there is no mechanism to study the upregulation of *MDR1* mRNA expression by DOX, and MPs were not isolated from the cell culture medium of drug-resistant strains and analysed for the components of MPs. In the literature, MPs have been reported to exhibit tissue selectivity during the transfer of drug-resistant proteins (Jaiswal et al., 2013) and carry nucleic acid components (Martins et al., 2013). Next, we will isolate MPs, analyse whether they contain *MDR1* mRNA or upregulate the relevant components of their expression, and further study the mechanism by which AqMDR cells upregulate *MDR1* mRNA expression.

## MATERIALS AND METHODS

### Main materials and reagents

The BIU-87 cell line was provided by the China Center for Type Culture Collection, Wuhan University, Wuhan, Hubei, People's Republic of China. The BIU-87/DOX cell line was provided by the Wuhan Union Hospital Urology Department, Huazhong University of Science and Technology, Wuhan, Hubei, People's Republic of China. RPMI-1640 medium and foetal bovine serum were purchased from Gibco. The CCK-8 kit and Hoechst 33342 were from the Beyotime Biotech (Haimen, Jiangsu, People's Republic of China). Mouse anti-human P-gp monoclonal antibody (sc-13131), mouse anti-human β-actin antibody (sc-47778), HRP (sc-2005), TR (sc-2781)-labelled goat anti-mouse IgG were purchased from Santa Cruz Biotechnology (Santa Cruz, CA, USA), TRIzol reagent and a Reverse Transcription Kit were purchased from Invitrogen, and PCR primer design and synthesis was completed by Invitrogen.

#### Cell culture

Cell lines BIU-87 and BIU-87/DOX were cultured in RPMI-1640 medium containing 10% foetal bovine serum, DOX at 1 μg ml^−1^ was added to the BIU-87/DOX medium to maintain its multidrug-resistance characteristics. All cells were cultured in an incubator containing 5% CO_2_ and 95% air humidity at 37°C.

#### AqMDR cells were observed by laser confocal microscopy

BIU-87 cells were inoculated into the lower chamber. Equal numbers of BIU-87/DOX cells were seeded in the transwell upper chamber. Cells in the lower chamber of the transwell (AqMDRs) were co-cultured for 24 h, 48 h, 72 h, or 96 h, incubated with a mouse anti-serum human P-gp monoclonal antibody. The cells were then incubated with TR-labelled goat anti-mouse IgG. Nuclear staining solution (Hoechst 33342) was added to the cells. The stained cells were treated with the appropriate amount of anti-fluorescence quenching sealant and observed under laser confocal microscopy after sealing.

#### Cell proliferation assay

BIU-87, BIU-87/DOX and co-cultured for 96 h AqMDR cells were dissociated into single-cell suspensions in medium without DOX. The cell concentration was adjusted to 1×10^5^ cells/ml, 200 μl (that is, 2×10^4^) was inoculated into a 24-well plate containing 800 μl of culture medium per well. The plate was taken out every 24 h, and three wells were randomly selected for cell counting to obtain the mean value, counted continuously for 7 days, and plotted on a growth curve. In addition, the above three cell lines were cultured in a single-cell suspension in medium containing 1 μg ml^−1^ of DOX, and a growth curve was plotted by counting these cells as well. The cell doubling time reference (Yin et al., 2013) was calculated using the following formula: doubling time=T×log 2/(log Nt−log No), where T is the culture time, and Nt and No are the number of cells at the beginning of culture and at the end of culture, respectively.

#### The 96hAqMDR cells were collected and subcultured in two groups

One group of cells was treated with 1 μg ml^−1^ DOX and culture was continued, without adding the drug to the other group. The number of passages was recorded. The treated and untreated AqMDR cells were transferred to the fourth, eighth, sixteenth, and twentieth generations, and the cells passaged to the twentieth generation were frozen for 1 month and then resuscitated (AqMDR after resuscitation) for subsequent experiments.

#### The CCK-8 method was used to detect the resistance index (RI) of AqMDR cells treated with different DOX concentrations

The different treatment groups of BIU-87, BIU-87/DOX and AqMDR cells inoculated into 96-well plates at a concentration of 3×10^3^ cells per well. After culture for 24 h, the cells were cultured with DOX at different concentrations (20 μg ml^−1^, 15 μg ml^−1^, 10 μg ml^−1^, 5 μg ml^−1^, 1 μg ml^−1^, 0.5 μg ml^−1^, 0.1 μg ml^−1^). Base 100 μl, and there were three replicate wells for each concentration, three additional wells of untreated cells and three medium-only wells (zero wells) for each cell type. After continuing to culture for 48 h, 10 μl of CCK-8 was added, the cells were incubated at 37°C for 4 h in the dark, and the A value was measured at the dual wavelength of the microplate reader. Inhibition rate=1−(average A value of the drug group−zero well)/(average A value of the unmedicated group−zero well), and the half-inhibitory concentration (IC50) of DOX was calculated by the Bliss method (Shi et al., 2006). RI=IC50 of sensitive cell/IC50 of resistant cells.

#### Western blot analysis of P-gp expression levels

BIU-87, BIU-87/DOX and AqMDR cells of different concentrations were collected and total protein was extracted as described previously (Zhou et al., 2013). The luminescent liquid was applied to the PVDF membrane, and placed in a gel imager (ChemiDoc XRS, Bio-Rad, USA) for exposure imaging. β-actin was used as an internal reference. The ECL luminescence results were recorded and analysed using Quantity One software, and the expression level of P-gp was analysed by the ratio of the P-gp and β-actin gray values.

#### RT-PCR detection of *MDR1* mRNA expression

BIU-87, BIU-87/DOX and AqMDR cells that underwent different treatments were collected, and total RNA was extracted by the TRIzol method. cDNA synthesis and PCR amplification were performed according to kit instructions. The upstream primer was 5′-CCCATCATTGCAATAGCAGG-3′, the downstream primer was 5′-GTTCAAACTTCTGCTCCTAG-3′, and the amplified product was 157 bp. β-actin was used as the internal control, and the upstream primer was 5′-TCCTGTGGCATCCACGAAACT-3′, the downstream primer was 5′-GAAGCATTTGCGGTGGACGAT-3′, and the amplified product was 314 bp. The reaction conditions were as follows: pre-denaturation at 94°C for 5 min; denaturation at 94°C for 30 s, annealing at 55°C for 30 s, extension at 72°C for 30 s, amplification for 35 cycles; extension at 72°C for 10 min; storage at 4°C. The PCR product was electrophoresed on a 2% agarose gel, and the product size was determined using DNA Marker as a standard molecular weight. The electrophoresis results were analysed using Quantity One Analysis software, and the expression level of MDR1 mRNA was analysed by the ratio of the gray values of MDR1 and β-actin.

### Statistical analysis

Statistical analysis was performed on the data using SPSS 20.0 software. The data in this experiment are continuous variables, expressed as the mean±standard deviation 
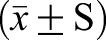
, and were compared using one-way analysis of variance. For differences between groups, the Bonferroni method was further used to compare multiple sample means for data that conformed to a normal distribution and had homogeneity of variance. Two-variable correlation analysis was performed using Spearman correlation analysis. *P*<0.05 indicated that a difference was statistically significant.
